# Network Pharmacology and Molecular Docking Elucidate the Pharmacological Mechanism of the OSTEOWONDER Capsule for Treating Osteoporosis

**DOI:** 10.3389/fgene.2022.833027

**Published:** 2022-02-28

**Authors:** Jiashuang Fan, Jianli Zhou, Zhuan Qu, Hangya Peng, Shuhui Meng, Yaping Peng, Tengyan Liu, Qiu Luo, Lifen Dai

**Affiliations:** ^1^ Department of Internal Medicine, The Second Affiliated Hospital of Kunming Medical University, Kunming, China; ^2^ Department of Internal Medicine, Yunnan Fuwai Cardiovascular Hospital, Kunming, China; ^3^ Medical School, Kunming Medical University, Kunming, China; ^4^ Department of Internal Medicine, The Affiliated Hospital of Yunnan University, Kunming, China

**Keywords:** network pharmacology, molecular docking, OSTEOWONDER capsule, osteoporosis, WGCNA

## Abstract

**Background:** Osteoporosis (OP) is a serious and common bone metabolic disease with bone mass loss and bone microarchitectural deterioration. The OSTEOWONDER capsule is clinically used to treat OP. However, the potential regulatory mechanism of the OSTEOWONDER capsule in treatment of OP remains largely unknown.

**Methods:** The bioactive compounds of herbs and their targets were identified using the Traditional Chinese Medicine Systems Pharmacology Database and Analysis Platform (TCMSP) database. The speculative targets of OP were screened out based on GeneCards, DisGeNET, and Online Mendelian Inheritance in Man (OMIM) databases. The gene modules and hub genes of OP were identified using a weighted gene co-expression network analysis (WGCNA). Then, an herb-compound-target network was constructed based on the above analyses. The biological function of targets was subsequently investigated, and a protein–protein interaction (PPI) network was constructed to identify hub targets of OP. Finally, molecular docking was performed to explore the interaction between compounds and targets.

**Results:** A total of 148 compounds of eight herbs and the corresponding 273 targets were identified based on the TCMSP database. A total of 4,929 targets of OP were obtained based on GeneCards, DisGeNET, and OMIM databases. In addition, six gene modules and 4,235 hub genes of OP were screened out based on WGCNA. Generally, an herb-compound-target network, including eight herbs, 84 compounds, and 58 targets, was constructed to investigate the therapeutic mechanism of the OSTEOWONDER capsule for OP. The biofunction analysis indicated 58 targets mainly associated with the bone metabolism, stimulation response, and immune response. EGFR, HIF1A, MAPK8, IL6, and PPARG were identified as the hub therapeutic targets in OP. Moreover, the interaction between EGFR, HIF1A, MAPK8, IL6, PPARG, and the corresponding compounds (quercetin and nobiletin) was analyzed using molecular docking.

**Conclusion:** Our finding discovered the possible therapeutic mechanisms of the OSTEOWONDER capsule and supplied the potential therapeutic targets for OP.

## Introduction

Osteoporosis (OP) is a systemic skeletal disease that is defined by a low bone mineral density (BMD) and T score, which is a standard deviation (SD) that represents the measured value of BMD varying from average or mean BMD of a healthy young adult (Johnston and Dagar, 2020). OP characterizes by a low bone mass and microarchitectural deterioration in the bone tissue and eventually induces bone fragility and fracture (Ensrud and Crandall, 2017). OP is a serious health problem that affects 200 million people worldwide and threatens 60.2 million older adults in China (Cotts and Cifu, 2018; Zeng et al., 2019). Unfortunately, OP not only has high incidence in the aging population but also majorly affects postmenopausal women, with approximately 10% of the world population and 30% of postmenopausal women suffering from OP (Alibasic et al., 2013; Zhu and Prince, 2015; Brown, 2017). The emergence of OP involves a number of factors, including genetic and environmental factors (Bijelic et al., 2016) as well as lifestyle, which has been recently demonstrated as an important factor contributing to OP in postmenopausal women (Zhu and Prince, 2015). In the past decades, several strategies have been used for prevention and treatment of OP, including anti-osteoporotic drugs (hormonal therapy, bisphosphonates, denosumab), anabolic agents (teriparatide), dual-action drugs (romosozumab), and strontium ranelate (Gatti and Fassio, 2019). However, although many therapeutic strategies have been used in clinics, many high-risk individuals remain without adequate treatment. The restricted and continued long treatment times usually cause treatment failure (Compston et al., 2019). Therefore, the challenges for the future are improving comprehensive nursing for OP patients and exploring more safe, simple, and effective medicines for OP treatment.

In recent years, traditional Chinese medicine (TCM) has been widely used for intractable disease treatment and has emerged with advantages of safety and effectiveness (Wang et al., 2018; Liu et al., 2020). For example, the combination of TCM with hepatic targeted drug delivery systems has been used for liver disease treatment (Ma et al., 2019). In addition, the TCM formula Lily Bulb and Rehmannia Decoction has been used for depression treatment (Chi et al., 2019). TCM formulas also show a good efficiency in treatment of Parkinson’s disease (Han et al., 2017), diabetes mellitus (Wang et al., 2020a), and idiopathic pulmonary fibrosis (Zhang et al., 2021). Moreover, TCM has been used as adjunctive therapy for improving the clinical outcome for cancer patients (Liao et al., 2017; Xiang et al., 2019; Zhai et al., 2019; Wang et al., 2020b). Some Chinese herbs and TCM formulas, such as Rehmanniae Radix (Liu et al., 2017a), Bushen-Jianpi-Huoxue decoction (Zhang et al., 2017), QiangGuYin (Shi et al., 2017), and Xianlinggubao capsules (Bao et al., 2020), have exhibited efficiency against OP.

The OSTEOWONDER capsule, also known as Heng-Gu-Gu-Shang-Yu-He-Ji and osteoking, is a TCM that originates from the Yunnan Province of China (Hu et al., 2005; Zhao et al., 2006) and has been approved by the Chinese State Food and Drug Administration in 2002 (Ling et al., 2021). The OSTEOWONDER capsule is comprised of Chenpi (*Citrus Reticulata Reticulatae*), Huangqi (*Hedysarum Multijugum* Maxim.), Renshen (*Panax Ginseng* C. A. Mey.), Honghua (*Carthamus tinctorius* L.), Sanqi (*Radix Notoginseng*), Duzhong (*Eucommiae Cortex*), Yangjinhua (*Daturae Flos*), Zuandifeng (*Schizophragma integrifolium*), and Biejia (*Trionycis carapace*). Previous studies have demonstrated that osteoking exerts anti-OP effects in OP rat models (Sun et al., 2020a; Sun et al., 2020b). In addition, it has been found that osteoking anti-OP may be through reduction of reactive oxygen species (Qin et al., 2019) or regulating rat bone marrow mesenchymal stem cell osteogenic and adipogenic differentiation (Yu et al., 2019). Nevertheless, the anti-osteoporotic pharmacological mechanism of osteoking remains unclear.

In the present study, a network pharmacology and bioinformatics analysis based on Gene Expression Omnibus (GEO) was utilized to explore the potential mechanism of the OSTEOWONDER capsule in the treatment of OP. Our finding provided the theoretical basis and evidence for OP treatment by the OSTEOWONDER capsule.

## Materials and Methods

### Screening the Bioactive Compounds and the Corresponding Targets From the OSTEOWONDER Capsule

The active compounds of the OSTEOWONDER capsule were derived from the Traditional Chinese Medicine Systems Pharmacology Database and Analysis Platform (TCMSP, https://tcmspw.com/index.php), which is a public and special traditional Chinese medicine system pharmacology database and analysis platform (Ru et al., 2014). The potential bioactive compounds of eight Chinese herbs, Chenpi, Huangqi, Renshen, Honghua, Sanqi, Duzhong, Yangjinhua, and Zuandifeng (Guang, 2009), were identified according to the oral bioavailability (OB) value ≥30% and drug-likeness (DL) ≥ 0.18. OB refers to the speed and extent of drug entering the circulatory system of the body. In addition, the OB value is represented as the percentage of the oral dose of drug entering the circulatory system. DL is a term that has been used to rationalize how physicochemical properties influence the molecular behavior *in vivo*. In addition, the corresponding targets of the bioactive compounds of the OSTEOWONDER capsule were identified based on the TCMSP database.

### Identification of the Targets of Osteoporosis

The name of osteoporosis was queried in GeneCards (https://www.genecards.org/) (Stelzer et al., 2016), the DisGeNET database (https://www.disgenet.org/home/) (Piñero et al., 2017; Piñero et al., 2020), and the Online Mendelian Inheritance in Man (OMIM) database (https://omim.org/), and the potential targets of OP were obtained.

### Data Processing and Weighted Gene Co-expression Network Analysis

Gene expression data and the corresponding clinical information of the GSE56815 dataset was downloaded from the Gene Expression Omnibus (GEO, https://www.ncbi.nlm.nih.gov/gds), which includes 40 OP and non-OP female blood monocyte samples. The platform of the GSE56815 dataset is the [HG-U133A] Affymetrix Human Genome U133A Array.

WGCNA is an efficient method to identify the clusters of highly correlated genes and to summarize such clusters using the module eigengene or an intramodular hub gene, so this is widely used to identify candidate biomarkers or therapeutic targets and analyze the connection between modules and the specific traits and phenotypes (Liu et al., 2017b; Cai et al., 2020). A WGCNA R package was used to perform the weighted correlation network construction and analysis (Langfelder and Horvath, 2008). First, all samples were clustered according to the gene co-expression similarity and the outliers were removed. Second, the power function “pickSoftThreshold” was used to screen the power parameter using a gradient method, which ranges from 1 to 20. An optimal soft threshold of 6 was selected as it met the degree of independence of 0.85 with the minimum power value. Finally, the adjacency matrix was transformed into the topological overlap matrix (TOM). Genes were assigned into different gene modules according to the TOM-related dissimilarity measure and the soft-thresholding setting. In addition, the numbers of gene modules were obtained according to the dissimilarity and the criterion of dynamic tree cutting with the minimal module size as 30 genes.

### Identification of Key Modules Correlating to Clinical Traits

After the modules were identified, the correlation between module eigengenes (ME) and clinical traits was investigated to identify the interest modules, which were significantly associated with clinical traits.

The modules significantly correlated with the clinical traits were identified as the OP-related modules in this study. Gene significance (GS) represents the correlation between gene expression and each trait, and module membership (MM) represents the correlation between gene expression and each module eigengene. Therefore, the correlation of module-clinical traits was confirmed by correlation between GS and MM. A correlation of module-clinical traits more than 0.3 served as the threshold to identify the interest modules in this study.

### Construction of the Herb–Compound–Target Network

The common genes were obtained by overlapping the targets of active compounds, targets of OP, and hub genes from key modules. Subsequently, a drug–bioactive compound–target network was constructed according to the eight herbs, 84 bioactive compounds, and 58 common genes using Cytoscape software version 3.7.2.

### GO and KEGG Pathway Enrichment Analysis

GO annotation and KEGG pathway enrichment analysis were performed using the clusterProfiler R package with adjusted-*P* value <0.05. GO annotation includes biological process (BP), molecular functions (MF), and cellular components (CC) terms.

### Protein–Protein Interaction Network Construction

In order to identify the interaction between the common genes, a PPI network was constructed using STRING (https://string-db.org/) with the Confidence of 0.4 and then visualized using Cytoscape version 3.7.2. Moreover, the potential hub targets and key sub networks were identified using “cytohbba” in Cytoscape according the Clustering Coefficient algorithm (Peng et al., 2021).

### Validation of the Expression of the Hub Targets

We explored the expression levels of five hub targets in different tissues based on Human Protein Atlas (HPA, https://www.proteinatlas.org/). In addition, we also investigated the correlation between the expression levels of five hub targets and clinical traits of OP using the Kruskal–Wallis test based on the GSE56815 dataset.

### Molecular Docking

Molecular docking is an important method which is used for drug design and discovery based on the interaction between the drug molecule and receptor. Molecular docking can be used to identify novel compounds and predict the ligand–target interaction at a molecular level or delineate structure–activity relationships (Pinzi and Rastelli, 2019). The bases of the molecular interaction and stable combination between molecules are spatial and energy matchings. Calculation of geometric matching was performed by grid computing and fragment growth, and energy calculation was performed by simulated annealing and genetic algorithms. AutoDock Vina is a common open-source and free software tool which is widely used for molecular docking. AutoDock Vina supports the AutoDock scoring function, simultaneous docking of multiple ligands, and a batch mode for docking a large number of ligands (Nguyen et al., 2020; Eberhardt et al., 2021). Here, we first obtained the protein structure of five hub proteins from the PDB database, then removed the existing water molecules and small molecules, and subsequently hydrogenated and calculated charges using AutoDock Tools. Second, we downloaded the structures of bioactive compounds and then checked the charge balance and rotatable bonds of small molecules. Third, the range of butt boxes was confirmed according to the receptor active center. Fourth, the semi-flexible docking of receptors and ligands was performed using AutoDock Vina by accepting the default settings, and the docking structures with the lowest binding free energy were output. The absolute values of negative values indicate the greater energy values. Finally, the docking structures were visualized and beautified using PyMol software (Yuan et al., 2016).

## Results

### Screening of Active Compounds and Potential Targets of the OSTEOWONDER Capsule

**FIGURE 1:**
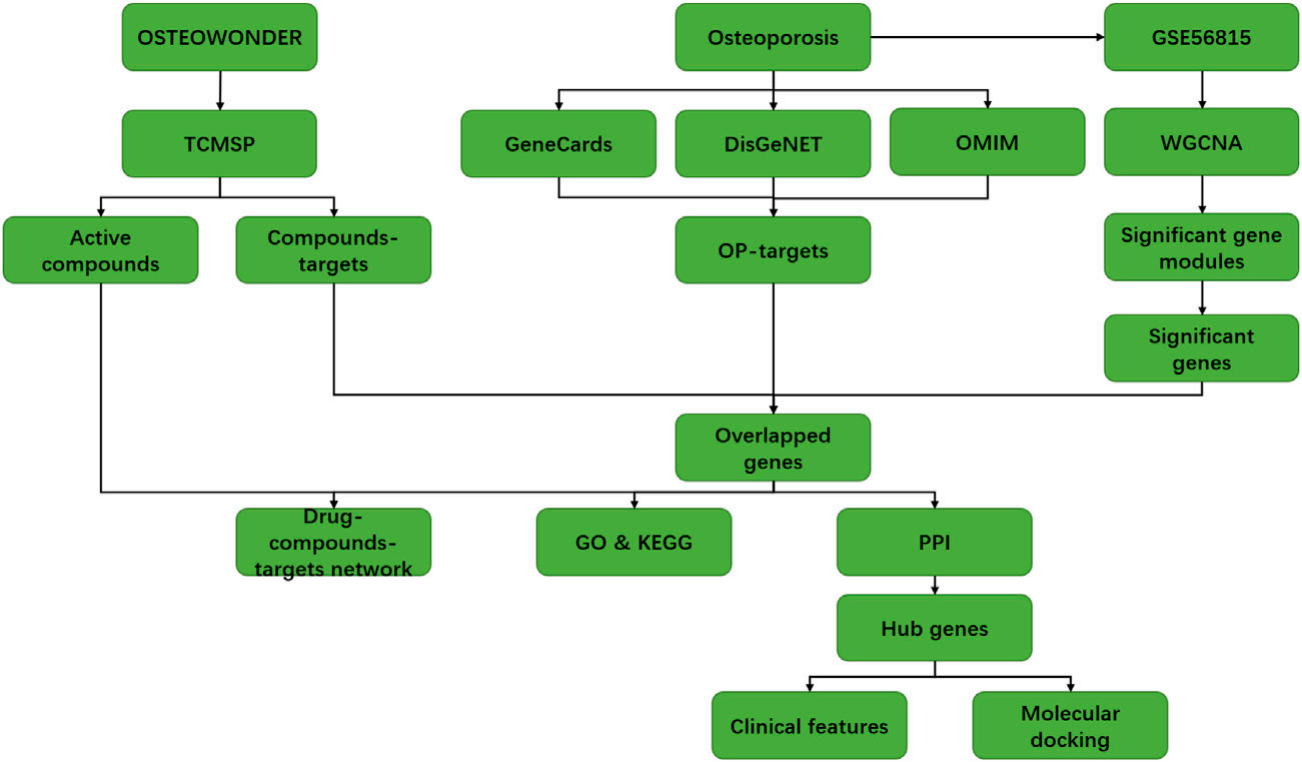
Flow diagram of the present study to investigate the potential mechanism of the OSTEOWONDER capsule in the OP treatment.

Considering the therapeutic effects of the OSTEOWONDER capsule on OP, we employed the system network pharmacology and WGCNA to explore the potential molecular mechanism of the OSTEOWONDER capsule in OP. The workflow of this study is shown in Figure 1. First of all, according to TCMSP database retrieval, a total of 917 compounds corresponding to seven herbs were obtained from the TCMSP database (Table 1). Thereafter, 148 bioactive compounds were screened based on the parameter of OB ≥ 30% and DL ≥ 0.18 (Table 1, Supplementary Table S1). In addition, we also predicated the targets of these compounds based on TCMSP, and we obtained a total of 1,153 targets of compounds (Table 2). Of these targets, 273 independent targets were obtained by removing duplication (Supplementary Tables S2, S3).

**TABLE 1:** Bioactivation compounds of the OSTEOWONDER capsule in TCMSP.

Herb ingredients	Latin name	Total number of compounds	Number of compounds (OB ≥ 30%, DL ≥ 0.18)
Chenpi	*Citrus Reticulata Reticulatae*	63	5
Huangqi	*Hedysarum Multijugum Maxim*	87	20
Renshen	*Panax Ginseng C. A. Mey*	190	22
Honghua	*Carthamus tinctorius L*	189	22
Sanqi	*Radix Notoginseng*	119	8
Duzhong	*Eucommiae Cortex*	147	28
Yangjinhua	*Daturae Flos*	106	27
Zuandifeng	*Schizophragma integrifolium*	16	16

**TABLE 2:** Targets of active compounds in TCMSP.

Herb ingredients	Latin name	Target number
Chenpi	*Citrus Reticulata Reticulatae*	63
Huangqi	*Hedysarum Multijugum Maxim*	191
Renshen	*Panax Ginseng C. A. Mey*	101
Honghua	*Carthamus tinctorius L*	200
Sanqi	*Radix Notoginseng*	173
Duzhong	*Eucommiae Cortex*	198
Yangjinhua	*Daturae Flos*	185
Zuandifeng	*Schizophragma integrifolium*	42

### Identification of the Potential Targets of Osteoporosis

Next, we screened OP-related genes based on the online databases. Unsurprisingly, a total of 4,613 targets of OP were identified from the GeneCards database (Supplementary Table S4), 1,098 targets of OP were identified from the DisGeNET database (Supplementary Table S5), and 197 targets of OP were screened from the OMIM database (Supplementary Table S6). A total of 4,929 targets of OP were obtained based on GeneCards, DisGeNET, and OMIM databases (Supplementary Table S7).

### Construction of WGCNA

**FIGURE 2:**
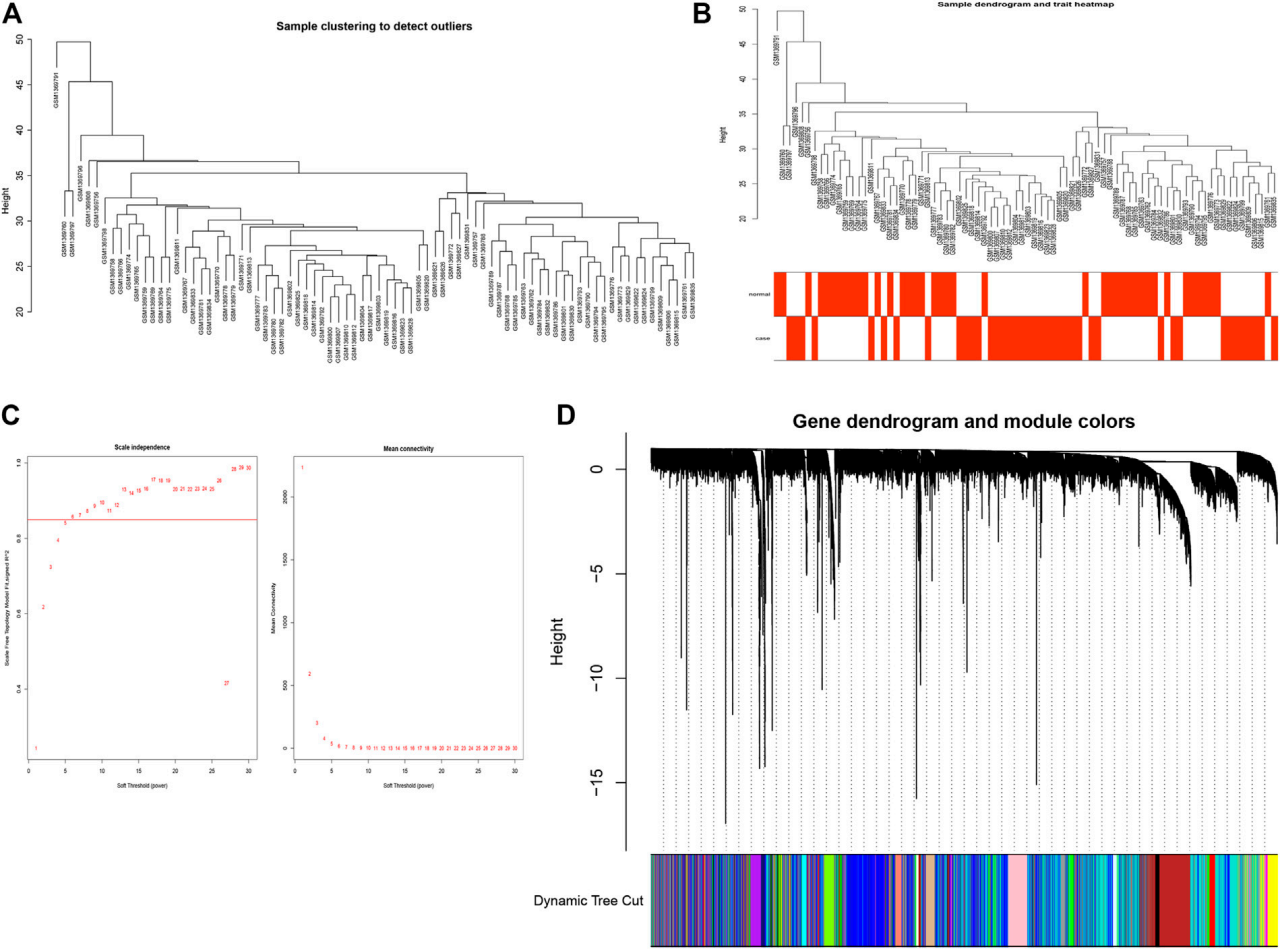
Construction of WGCNA. **(A)** Clustering dendrogram of samples **(B)** Clustering dendrogram of samples with trait heatmap. **(C)** Analysis of the scale-free index for various soft-threshold powers (β). Left panel, *X*-axis represents a function of soft-threshold power, and *Y*-axis represents the scale-free fit index. Right panel, *X*-axis represents a function of soft-threshold power, and *Y*-axis represents the mean connectivity (degree). **(D)** Clustering dendrogram of genes based on the measurement of dissimilarity (1-TOM).

In order to identify the comprehensive regulatory networks for OP, we employed a WGCNA to determine a network correlated to OP. In this study, a total of 13,230 genes in 80 samples were used to construct the co-expression network. Moreover, the clustering results of samples are shown as Figures 2A,B; these results indicated the high quality of clustering, and therefore, no outliers were removed in these samples. A soft-threshold power of 6 was used to obtain the approximate scale-free topology fit index (signed *R* ^2^) > 0.85 and the lowest power (Figure 2C). As a result, 13,230 genes were clustered into a total of 23 modules with the minimal module size as 30 genes using the average linkage hierarchical clustering algorithm (Figure 2D, Supplementary Table S8).

### Identification of the Hub Modules and Genes for OP via WGCNA

**FIGURE 3:**
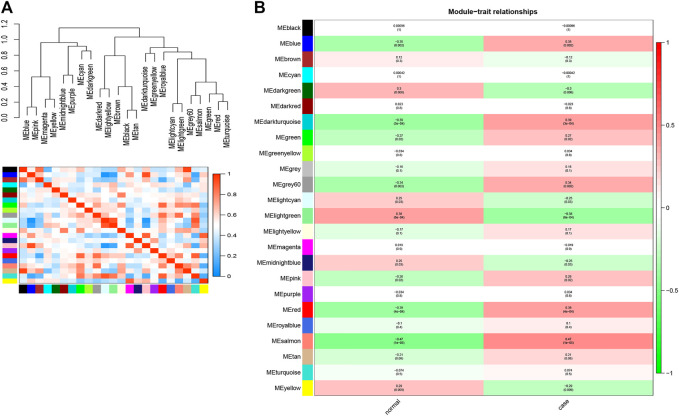
Identification of the hub modules and genes for OP via WGCNA. **(A)** Eigengene dendrogram and the heatmap of eigengene adjacency. **(B)** Heatmap of the correlation between module eigengenes and clinical traits of OP.

After construction of the WGCNA, we further explored the interaction among these modules. As shown in Figure 3A, the eigengene dendrogram and the eigengene adjacency heatmap indicated that 23 modules could be divided into several subgroups, suggesting the differences of correlation between different modules. Subsequently, we detected the correlation between modules and clinical traits according to the module-trait association analysis. Focusing on the case trait (i.e., OP), a total of six modules, including the blue module (|cor| > 0.3 and *p* < 0.05), darkturquoise module (|cor| > 0.39 and *p* < 0.05), grey60 module (|cor| > 0.34 and *p* < 0.05), lightgreen module (|cor| > 0.38 and *p* < 0.05), red module (|cor| > 0.39 and *p* < 0.05), and salmon module (|cor| >0.47 and *p* < 0.05), were identified to have the highest association with OP (Figure 3B). Moreover, a total of 3,410, 41, 94, 66, 524, and 118 genes were obtained from blue, darkturquoise, grey60, lightgreen, red, and salmon modules, respectively (Supplementary Table S9). Therefore, a total of 4,235 hub genes were identified based on WGCNA.

### Construction of the Herb–Compound–Target Network

**FIGURE 4:**
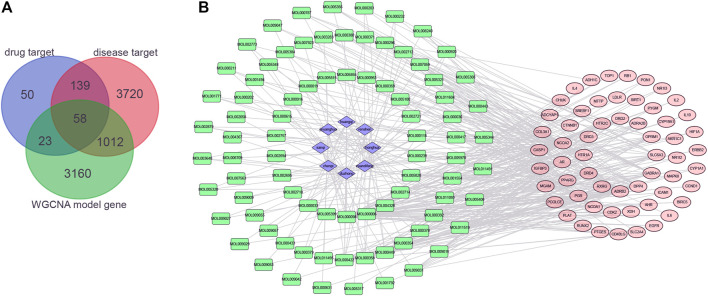
Construction of the herb–compound–target network. **(A)** Venn plot indicates the common targets among drug targets from the TCMSP database; targets of OP from GeneCards, DisGeNET, and OMIM databases; and hub genes from WGCNA. **(B)** Herb–compound–targets network. Blue rhombus represents herbs, green rectangle represents compounds, and red ellipse represents targets of OP.

Generally, 58 potential targets of OP were identified by overlapping the 273 targets of compounds, 4,929 targets of OP, and 4235 OP-related hub genes (Figure 4A; Supplementary Table S10). To further investigate the effectiveness of the OSTEOWONDER capsule to prevent OP, which depends on the synergistic effects of compounds and their targets, an herb–compound–target network was constructed in this study based on eight herb ingredients, 84 bioactive compounds, and 58 common targets (Figure 4B, Supplementary Table S11). The network included 150 nodes (eight herb ingredients, 84 bioactive compounds, and 58 common targets) and 422 edges.

### Biofunction Analysis of Targets

**FIGURE 5:**
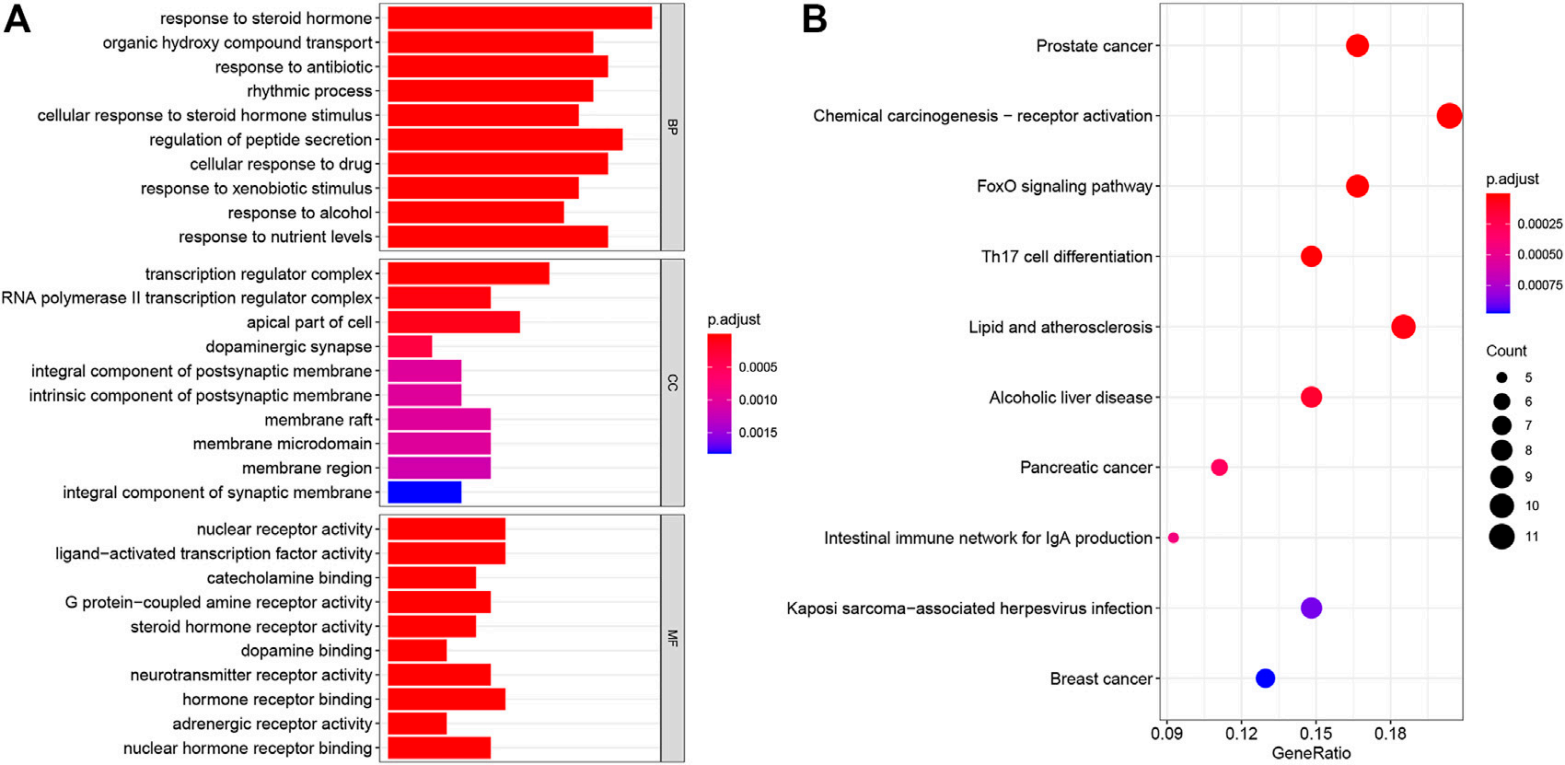
Biofunction analysis of targets. **(A)** Bar plot indicates the GO enrichment analysis of the common targets, including top 10 BP, top 10 CC, and top 10 MP terms. **(B)** Bubble plot indicates the top 10 pathways by KEGG pathway enrichment analysis of the common targets.

To further investigate the potential mechanism of the OSTEOWONDER capsule for OP, GO and KEGG enrichment analyses were conducted based on 58 targets. We found these genes enriched in 778 BP terms, 34 CC terms, and 48 MF terms (Supplementary Table S12). The top 10 BP terms are shown in Figure 5A, including response to the steroid hormone, organic hydroxy compound transport, response to antibiotics, the rhythmic process, cellular response to steroid hormone stimuli, regulation of peptide secretion, cellular response to the drug, response to xenobiotic stimuli, response to alcohol, and response to nutrient levels. In addition, the top 10 CC terms included the transcription regulator complex, RNA polymerase Ⅱ transcription regulator complex, the apical part of the cell, dopaminergic synapse, the integral component of the postsynaptic membrane, the intrinsic component of the postsynaptic membrane, the membrane raft, the membrane microdomain, the membrane region, and the integral component of the synaptic membrane (Figure 5A). Moreover, the top 10 MF terms concentrated on nuclear receptor activity, ligand-activated transcription factor activity, catecholamine binding, G-protein-coupled amine receptor activity, steroid hormone receptor activity, dopamine binding, neurotransmitter receptor activity, hormone receptor binding, adrenergic receptor activity, and nuclear hormone receptor binding (Figure 5A). Additionally, these genes were enriched in 73 KEGG pathways (Supplementary Table S13), and the top 10 pathways were screened according to adjusted-*P* value <0.05, including prostate cancer, chemical carcinogenesis-receptor activation, the Foxo signaling pathway, Th17 cell differentiation, lipid and atherosclerosis, alcoholic liver disease, pancreatic cancer, the intestinal immune network for IgA production, Kaposi sarcoma-associated herpesvirus infection, and breast cancer pathways (Figure 5B). These results suggested that the OSTEOWONDER capsule for OP treatment might regulate the bone metabolism, stimulation response, and immune response.

### Construction of the PPI Network and Screening Hub Targets

**FIGURE 6:**
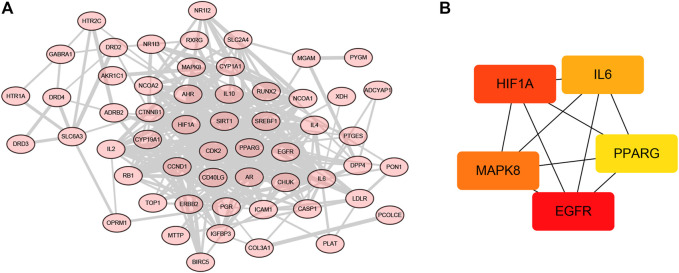
Construction of the PPI network and screening hub targets. **(A)** PPI network of 58 common targets. **(B)** Subnetwork of the PPI network.

In order to clarify the potential mechanisms of 58 targets of the OSTEOWONDER capsule for OP treatment, we constructed a PPI network based on interactions among 58 targets. As shown in Figure 6A, Supplementary Table S14, a PPI network included 56 nodes and 310 edges were constructed according to the confidence of 0.4. We further identified the subnetwork and hub targets from the PPI network using the cytohubba function. As shown in Figure 6B, Supplementary Table S15, a subnetwork was identified, including five nodes and 10 edges. Moreover, EGFR, HIF1A, MAPK8, IL6, and PPARG were identified as the hub targets in the OSTEOWONDER capsule for OP treatment.

### Exploration of the Expression of Hub Targets

**FIGURE 7:**
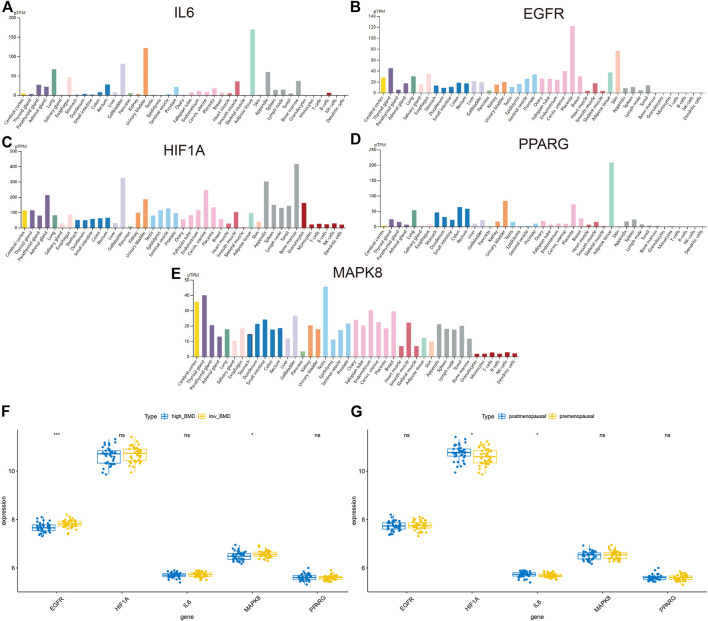
Exploration of the expression of hub targets. **(A–E)** Histograms of IL6, EGFR, HIF1A, PPARG, and MAPK8 in different tissues based on the HPA database. **(F)** Histograms showing the correlation between BMD and IL6, EGFR, HIF1A, PPARG, and MAPK8. **(G)** Histograms showing the correlation between pre-/postmenopausal and IL6, EGFR, HIF1A, PPARG, and MAPK8.

In order to analyze the functions of hub targets in OP, we first detected the expression of five hub targets in the HPA database. The results indicated that IL6, HIF1A, and MAPK8 expressed in the bone marrow (Figures 7A–E). We also detected the association between these gene expression and clinical traits, including bone mineral density (BMD) and female pre-/postmenopause, as shown in Figures 7F,G and Supplementary Table S16, EGFR and MAPK8 associated with low BMD, HIF1A, and IL6 were associated with postmenopausal patients.

### Bioactive Compound–Target Docking

**FIGURE 8:**
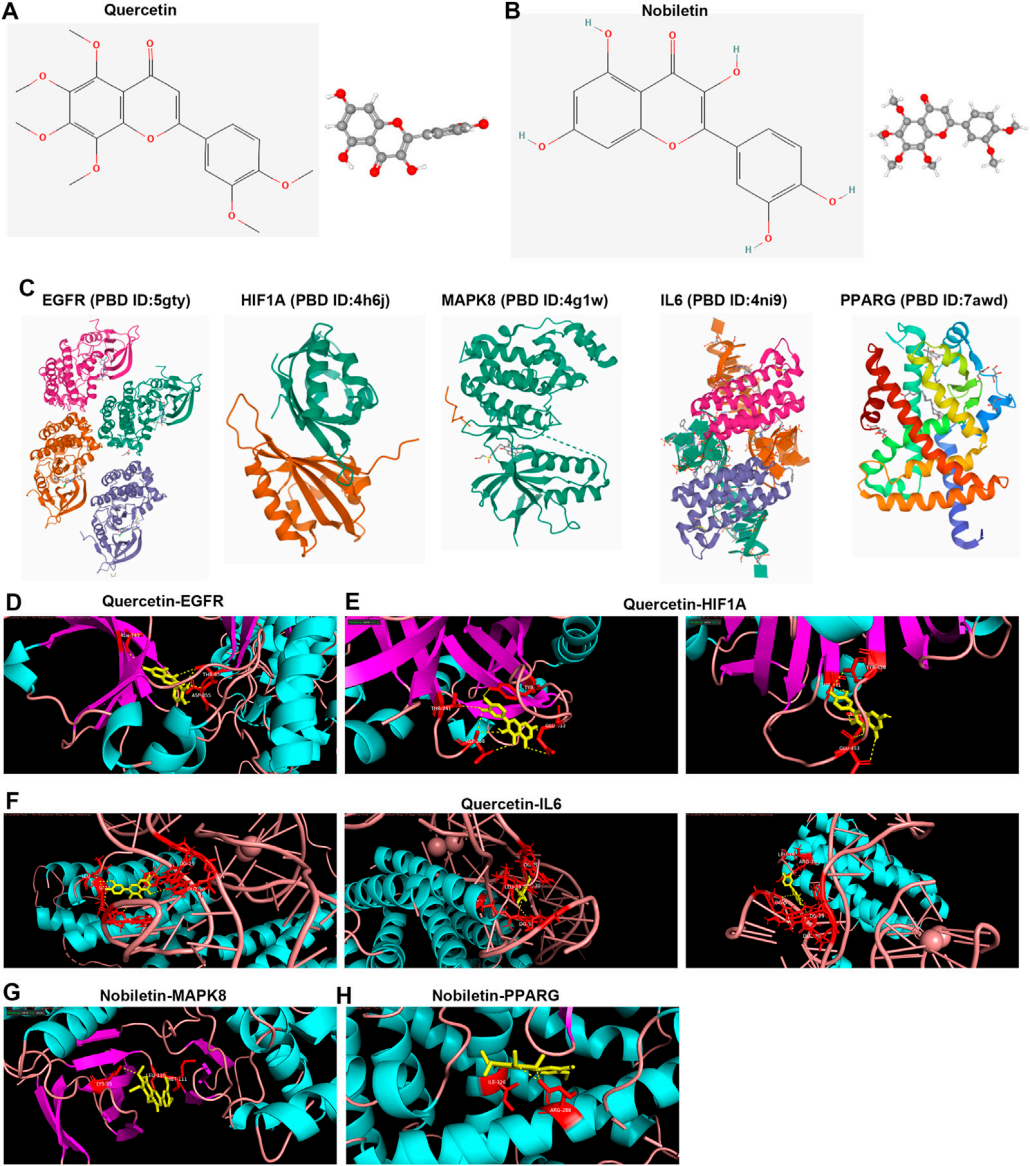
Bioactive compound–target docking. **(A,B)** Chemical structures of quercetin and nobiletin. **(C)** Tertiary structure of proteins, including EGFR, HIF1A, MAPK8, IL6, and PPARG. **(D,H)** Molecular docking of quercetin–EGFR, quercetin–HIFIA, quercetin–IL6, nobiletin–MAPK8, and nobiletin–PPARG. The red sticks represent amino acid residues, and yellow dotted lines represent hydrogen bonds.

We further performed compound–target docking to determine the interaction of the targets and their corresponding compounds. According to the herb ingredient–compound–target network, we found five hub targets mainly interacting with quercetin and nobiletin (Table 3). Therefore, we analyzed the interaction relationship between these targets and compounds. Before performing molecular docking, the structures of quercetin and nobiletin were downloaded from PubChem (Figures 8A,B), and the tertiary structures of EGFR, HIF1A, MAPK8, IL6, and PPARG proteins were obtained from the PDB database (Figure 8C). Then, molecular docking was performed using AutoDock Vina and visualized using PyMol software. We found that quercetin targeted with ALA-743, ASP-855, and THR-854 residues of EGFR through hydrogen bonding (Figure 8D), and EGFR strongly targeted with quercetin (docking score = −9.1 kcal/mol). HIF1A interacted with quercetin by hydrogen bonding connecting quercetin and THR-241, ASP-238, THR-456, and GLU-453 residues (Figure 7E), and docking score = −7.7 kcal/mol. In addition, IL6 strongly interacted with quercetin through hydrogen bonding connecting quercetin and LEU-19, ARG-24, DG-5, DG-29, and DUZ-30 based on docking score = −10.8 kcal/mol (Figure 7F). MAPK8 also strongly interacted with nobiletin (docking score = −7.5 kcal/mol), and LYS-55, LEU-110, and MET-111 targeted with nobiletin through hydrogen bonding (Figure 7G). Moreover, nobiletin targeted with PPARG through hydrogen bonding connecting ILE-326, ARG-288, and nobiletin based on docking score = −8.1 kcal/mol (Figure 7H).

**TABLE 3:** bioactive compounds with targets.

Molecular ID	Compounds	Candidate targets
MOL000098	Quercetin	EGFR
MOL000098	Quercetin	HIF1A
MOL000098	Quercetin	IL6
MOL005828	nobiletin	MAPK8
MOL005828	nobiletin	PPARG

## Discussion

Although TCM treatment in China had a long history of thousands of years (Li and Weng, 2017), due to the lack of modern scientific and clinical evidence, and the absence of explicit molecular mechanisms and targets, TCM has been regarded as a complementary or alternative form of medicine in western countries, and this obstructs the worldwide application of TCM (Li et al., 2016; Wang et al., 2018). Artemisinin derives from the herbal *Artemisia annua*, which is a potent anti-malarial that was discovered by Tu Youyou. The discovery shocked the world and highlights the potential value of herbal medicine (Tu, 2016). In recent years, more and more Chinese herbals have been widely used for most disease treatments based on modern science and computer technology (Huang et al., 2019; Yang et al., 2021a; Niu et al., 2021). Network pharmacology is a powerful method that is increasingly applied for TCM research, including compounds of herb identification, targets of compound investigation, network construction, network analysis, and network verification (Luo et al., 2020).

The OSTEOWONDER capsule is a Yi ethnic medicine widely used for OP treatment (Jianming, 2020; Zhengying et al., 2021). However, the molecular mechanism of the OSTEOWONDER capsule in OP treatment remains unclear. The OSTEOWONDER capsule is comprised of Chenpi (*Citrus Reticulata Reticulatae*), Huangqi (*Hedysarum Multijugum* Maxim.), Renshen (*Panax Ginseng* C. A. Mey.), Honghua (*Carthamus tinctoriu*s L.), Sanqi (*Radix Notoginseng*), Duzhong (*Eucommiae Cortex*), Yangjinhua (*Daturae Flos*), Zuandifeng (*Schizophragma integrifolium*), and Biejia (*Trionycis carapace*). Here, we focused on the functions and regulatory mechanism of eight herbs. In this study, a total of 148 bioactive compounds and the corresponding 273 targets were identified based on TCMSP, and 4,929 targets of OP were obtained from GeneCards, DisGeNET, and OMIM databases. Moreover, six modules and 4,235 hub targets associated with OP were identified based on WGCNA. Then, 58 intersected genes were obtained by overlapping the above genes. A drug–compound–target network was constructed, which includes eight herbs, 84 bioactive compounds, and 58 targets.

The GO and KEGG pathway enrichment analyses indicated that the OSTEOWONDER capsule for OP treatment might regulate the bone metabolism, stimulation response, and immune response. For example, the biological process enrichment analysis indicated that these targets mainly associated to response to the steroid hormone, organic hydroxy compound transport, response to antibiotics, the rhythmic process, cellular response to steroid hormone stimulus, regulation of peptide secretion, cellular response to the drug, response to xenobiotic stimulus, response to alcohol, and response to nutrient levels. Steroid-induced OP is the most common form of secondary OP (Adami and Saag, 2019), and a previous study has indicated Rehmanniae radix anti-OP by intervening steroid hormone biosynthesis (Xia et al., 2019). Here, we found that the OSTEOWONDER capsule exerted anti-OP effects and was involved in response to the steroid hormone or steroid hormone stimuli. Besides, the nutrients and dietary patterns were significantly associated with OP (Muñoz-Garach et al., 2020), and OSTEOWONDER capsule anti-OP also regulated the biological process of response to nutrient levels. Besides, the molecular function was involved in nuclear receptor activity, ligand-activated transcription factor activity, catecholamine binding, G-protein-coupled amine receptor activity, steroid hormone receptor activity, dopamine binding, neurotransmitter receptor activity, hormone receptor binding, adrenergic receptor activity, and nuclear hormone receptor binding. In addition, KEGG pathway enrichment analysis revealed that these genes were mainly enriched in the Foxo signaling pathway and Th17 cell differentiation. The Foxo signaling pathway acts as an important role in regulating bone cell functions and bone health; for example, Foxo promotes normal skeletal development in chondrocytes but decreases bone resorption and formation in osteoclasts (Kim et al., 2018; Ma et al., 2020). It is known to us that regulatory T (Treg) cells and T helper 17 (Th17) cells play crucial roles in maintaining bone homeostasis, and Th17 cells promote osteoclast differentiation, which contribute to OP (Zhu et al., 2020). Maintaining the balance between Treg and Th17 cells may provide a novel promising strategy for treatment of OP (Yang et al., 2021b; Sapra et al., 2021).

In addition, we investigated the interaction relationship between these targets and identified hub targets by constructing the PPI network, and EGFR, HIF1A, MAPK8, IL6, and PPARG were identified as the hub targets in the network with the highest degree. Molecular docking was performed to mimic the connection between EGFR, HIF1A, MAPK8, IL6, and PPARG and two compounds (quercetin and nobiletin). Previous studies have demonstrated that EGFR, HIF1A, MAPK8, IL6, and PPARG exert important roles in OP progression; for example, downregulated EGFR promotes bone formation on the endosteal surface of the cortical bone (Liu et al., 2019). In addition, MAPK8 is involved in the remarkable bone loss (Li et al., 2018). However, HIF1A facilitates osteocyte-mediated osteoclastogenesis *in vitro* (Zhu et al., 2019). IL-6 exerts dual effects on the differentiation of osteoblasts and osteoclasts (Wang and He, 2020). Moreover, PPARG is essential for sclerostin production, which has been approved as a target for treating OP (Baroi et al., 2021). Here, we identified these genes as the key targets for OP treatment by the OSTEOWONDER capsule. Two compounds, quercetin and nobiletin, were also found to mainly target these genes.

Quercetin is one of the members of the naturally occurring flavonoid family, and is well known for its anti-oxidant, anti-inflammatory, and anti-tumor properties (Eid and Haddad, 2017; Tang et al., 2020). Surprisingly, quercetin exerts anti-OP by promoting osteogenic differentiation, anti-oxidant response, and anti-inflammatory properties (Wang et al., 2017; Yuan et al., 2018; Huang et al., 2020; Oh et al., 2020; Wang et al., 2021). Here, we found that quercetin has been used as an agent for OP by reducing EGFR, HIF1A, and IL6 expression. EGFR, HIF1A, and IL6 have also been demonstrated as the therapeutic targets of OP (Fayed et al., 2019; Luo et al., 2021; Xiong et al., 2021). Nobiletin, a polymethoxylated flavone, exhibits anti-oxidative, anti-inflammatory, and anti-cancer properties (Murakami et al., 2000; Hagenlocher et al., 2019; Feng et al., 2020). It has been found that nobiletin exerts the anti-OP effects in mice (Matsumoto et al., 2018; Wang et al., 2019). In the present study, we found that nobiletin exerted the anti-OP effects by targeting MAPK8 and PPARG.

## Conclusion

In summary, a network pharmacology approach was applied to identify bioactive compounds of the OSTEOWONDER capsule and the corresponding targets. In addition, we also systemically screened out the therapeutic targets of the OSTEOWONDER capsule on OP based on online databases and WGCNA. Our evidence indicated the potential mechanism by which the OSTEOWONDER capsule ameliorates multiple pathological features of OP with multiple targets and biological pathways. Our finding provided evidence supporting the clinical application of the OSTEOWONDER capsule in OP treatment.

## Data Availability

The datasets presented in this study can be found in online repositories. The names of the repository/repositories and accession number(s) can be found in the article/Supplementary Material.
