# Supplementary material for: Network Pharmacology and Molecular Docking Elucidate the Pharmacological Mechanism of the OSTEOWONDER Capsule for Treating Osteoporosis
Source: Front Genet. 2022 Feb 28;13:833027. doi: 10.3389/fgene.2022.833027 (PMC8918533; doi:10.3389/fgene.2022.833027)
Supplement: Supplementary file 2 [file DataSheet2.docx]

**Table 1 Bioactivation compounds of** **OSTEOWONDER capsule in** **TCMSP.**

| Herb ingredients | Latin name | Total number of compounds | Number of compounds (OB ≥ 30%, DL ≥ 0.18) |
| --- | --- | --- | --- |
| Chenpi | *Citrus Reticulata Reticulatae* | 63 | 5 |
| Huangqi | *Hedysarum Multijugum Maxim.* | 87 | 20 |
| Renshen | *Panax Ginseng C. A. Mey.* | 190 | 22 |
| Honghua | *Carthamus tinctorius L.* | 189 | 22 |
| Sanqi | *Radix Notoginseng* | 119 | 8 |
| Duzhong | *Eucommiae Cortex* | 147 | 28 |
| Yangjinhua | *Daturae Flos* | 106 | 27 |
| Zuandifeng | *Schizophragma integrifolium* | 16 | 16 |

**Table 2 Targets of active compounds in TCMSP.**

| Herb ingredients | Latin name | Target number |
| --- | --- | --- |
| Chenpi | *Citrus Reticulata Reticulatae* | 63 |
| Huangqi | *Hedysarum Multijugum Maxim.* | 191 |
| Renshen | *Panax Ginseng C. A. Mey.* | 101 |
| Honghua | *Carthamus tinctorius L.* | 200 |
| Sanqi | *Radix Notoginseng* | 173 |
| Duzhong | *Eucommiae Cortex* | 198 |
| Yangjinhua | *Daturae Flos* | 185 |
| Zuandifeng | *Schizophragma integrifolium* | 42 |

**Table 3 Bioactive compounds interact to targets.**

| Molecular ID | Compounds | Candidate targets |
| --- | --- | --- |
| MOL000098 | Quercetin | EGFR |
| MOL000098 | Quercetin | HIF1A |
| MOL000098 | Quercetin | IL6 |
| MOL005828 | nobiletin | MAPK8 |
| MOL005828 | nobiletin | PPARG |
